# Supplementary material for: A holistic approach for suppression of COVID-19 spread in workplaces and universities
Source: PLoS One. 2021 Aug 12;16(8):e0254798. doi: 10.1371/journal.pone.0254798 (PMC8360595; doi:10.1371/journal.pone.0254798)
Supplement: S1 File — (ZIP) [file pone.0254798.s001.zip › S1_File.docx]

# **Supporting information S1: Supplementary methods**

We present here a two-group compartmental model that accounts for transmission dynamics of SARS-CoV-2 [1-3] within and between two groups: (i) employees in the workplace (“the workplace”, denoted as “W”); (ii) non-employees (“the community”, denoted as “C”). The basic modeling framework rests on the following key assumptions (S1 Fig):

1. **FIXED WORKFORCE**: All individuals are either employees or non-employees.
2. **VARIABLE SPREAD**: The transmission rate in the workplace and in the community may differ.
3. **COMMON BIOLOGY**: The progression of the disease, including time to develop symptoms and time to recover, is the same among employees and non-employees.
4. **SHARED COMMUNITY**: Employees spend p% of their time at work and isolated from the non-employees in the community. During the remaining (100 - p)% of time, employees interact with both employees and non-employees in the community (though their interactions may quantitatively differ).

**Figure S1: Foundational principles for modeling disease transmission dynamics within and between the community and the workplace.**

**(a) FIXED WORKFORCE**: People in the workforce remain as “employees” during the entire simulation, and all other individuals are considered “non-employees”. **(b) VARIABLE SPREAD**: Transmission dynamics among employees may differ from transmission among non-employees. In the cartoon example shown here, taking panel (a) as the starting point, the 2 infected out of 72 non-employees have infected twice as many non-employees (4), whereas the 1 infected of 18 employees has infected only 1 additional employee (perhaps due to stricter social distancing in the workplace). **(c) COMMON BIOLOGY**: In this example, taking panel (b) as the starting point, a week after their infections have started, half of the infected individuals have recovered, irrespective of being an employee or not. **(d) SHARED COMMUNITY**: Outside of the workplace, employees and non-employees mix and allow for transmission of virus within this larger group.

These assumptions are translated into a dynamic, deterministic, two-group compartmental model that we call the “Community-Workplace” model. This model is composed of a SEPAYR (Susceptible - Exposed - Presymptomatic - Asymptomatic - sYmptomatic - Recovered) compartmental model for non-employees and a SEPAYDR (Susceptible - Exposed - Presymptomatic - Asymptomatic - sYmptomatic - Detected - Recovered) compartmental model for employees (Fig 1, S2 Table, and S1.1 - S1.16 Equations).

**S2 Table: Notation and formulae for parameters used in the “Community-Workplace” model.**

| **Model Concept** | **Notation** |
| --- | --- |
| Length of modeling simulation (days) | L |
| Time (timepoint of the simulation) | t |
| “Workplace” population size (i.e., number of employees) | N_W_ |
| “Community” population size (i.e., number of non-employees) | N_C_ |
| Initial disease prevalence in the community (at the beginning of the simulation) | prv_Ci_ |
| Initial disease prevalence in the workplace (at the beginning of the simulation) | prv_Wi_ |
| Proportion of time employees spend at work (interacting only among themselves) | p |
| Proportion of cases that develop symptoms (vs. being “asymptomatic”) | q |
| Proportion of non-cases that report symptoms each work-day | g |
| Average days it takes to develop infectiousness after infection-causing exposure to virus | Δ_infectious_ |
| Rate of development of infectiousness (= 1 / Δ_infectious_) | θ |
| Average days of being infectious. Equivalent to the average days taken to recover from onset of infectiousness. | Δ_recover_ |
| Rate of recovery from onset of infectiousness (= 1 / Δ_recover_) | ɣ**_I_** |
| Average days taken to develop symptoms after becoming infectious (for non-“asymptomatic”s) | Δ_symptoms_ |
| Rate of symptom development for infected individuals who become symptomatic (= 1 / Δ_symptoms_) | ƛ |
| Rate of recovery from onset of symptoms (= 1 / (Δ_recover_ - Δ_symptoms_)) | ɣ_Y_ |
| Rate of recovery from moving into asymptomatic compartment (= 1 / (Δ_recover_ - Δ_symptoms_)) | ɣ_A_ |
| Days required in isolation if tested positive | Δ_isolation_ |
| Rate of movement back to work after being detected (= 1 / Δ_isolation_) | ɣ_D_ |
| Average days of immunity after recovering | Δ_immunity_ |
| Rate of loss of immunity (= 1 / Δ_immunity_) | ⍺ |
| Basic virus reproduction number (i.e., the mean number of people in a fully susceptible population that are infected with SARS-CoV-2 by a single infected person) in the workplace | R0_W_ |
| Basic virus reproduction number (i.e., the mean number of people in a fully susceptible population that are infected with SARS-CoV-2 by a single infected person) in the community | R0_C_ |
| Transmission rate in the workplace (= R0_W_ * ɣ**_I_**) | β_W_ |
| Transmission rate in the community (= R0_C_ * ɣ**_I_**) | β_C_ |
| Proportion of the asymptomatic workforce population tested each day | 𝜏_A_ |
| Proportion of the symptomatic workforce population tested each day | 𝜏_Y_ |
| Test sensitivity (probability of an infected individual’s test being positive) | sens |
| Average number of tests required to return to work after infection (i.e., “testing out of isolation”) | h |

Under this idealized model, susceptible individuals (S_C_ and S_W_, for non-employees and employees, respectively) can be infected by either employees or non-employees (P_C_, A_C_, and Y_C_, or P_W_, A_W_, and Y_W_, respectively; see below). This is achieved using a variable p that defines the amount of time the employees spend isolated among themselves in the workplace. During this time, employees only interact with one another, and as such, infection can only be transmitted from employee to employee. Similarly, non-employees only interact with one another during this time. In the remaining time (1 - p), the employees and non-employees act as one population, allowing infection to be transmitted between employees and non-employees. Upon becoming infected, individuals move into an exposed state (E_C_ and E_W_, for non-employees and employees, respectively). While in the exposed state, the viral load is considered too low to be detectable, and the individual is modeled as not yet being infectious. Once the viral load increases sufficiently, the individual becomes infectious and would also return a positive result from a diagnostic test that has 100% sensitivity. This is a simplification we use here of the relationship between viral load and the probability of testing positive, though there is reason to believe that test sensitivity increases as viral load increases, rather than having a distinct threshold [4]. Infectious individuals fall into three compartments, presymptomatic (P_C_ or P_W_), asymptomatic (A_C_ or A_W_), and symptomatic (Y_C_ or Y_W_). Infected employees who test positive in the workplace testing program move into a detected compartment (D_w_). All infected individuals move to a recovered compartment (R_C_ or R_W_, for non-employees and employees, respectively) once viral load again drops to a non-infectious level; as noted above, for simplicity, this is equated with being below detectable levels for testing. This model assumes that symptomatic and asymptomatic individuals are equally infectious: for more detail on this assumption, see S2 below.

Using the notation defined in S2 Table, the specified dynamics of this two-group “Community-Workplace” compartmental model are governed by the system of differential equations given in S1.1 - S1.14 Equations. Note that the model used herein was implemented using discrete-time difference equations, but differential equations are shown here for clarity.

### **Non-employee SEPAYR:**

d**S_C_**/dt = - [p * β_C_ * S_C_ * (P_C_ + A_C_ + Y_C_) / N_C_] *(exposure from non-employees, during work hours)* **(S1.1)**

- [(1 - p) * β_C_ * S_C_* (P_C_ + A_C_ + Y_C_) / (N_C_ + N_W_)] *(exposure from non-employees, after work hours)*
- [(1 - p) * β_C_* S_C_* (P_W_ + A_W_ + Y_W_) / (N_C_ + N_W_)] *(exposure from employees in the community)*
- [⍺ * R_C_] *(loss of immunity)*

d**E_C_**/dt = [p * β_C_* S_C_ * (P_C_ + A_C_ + Y_C_) / N_C_ ] *(exposure from non-employees, during work hours)* **(S1.2)**

- [(1 - p) * β_C_* S_C_* (P_C_ + A_C_ + Y_C_) / (N_C_ + N_W_)] *(exposure from non-employees, after work hours)*
- [(1 - p) * β_C_* S_C_* (P_W_ + A_W_ + Y_W_) / (N_C_ + N_W_)] *(exposure from employees in the community)*
- [θ * E_C_] *(development of infectiousness)*

d**P_C_**/dt = [θ * E_C_] *(development of infectiousness)* **(S1.3)**

- [ƛ * P_C_ * (1 - q)] *(classification as persistently asymptomatic)*
- [ƛ * P_C_ * q] *(development of symptoms)*

d**A**_C_/dt = [ƛ * P_C_ * (1 - q)] *(classification as persistently asymptomatic)* **(S1.4)**

- [ɣ_A_ * A_C_] *(recovery from asymptomatic)*

d**Y**_C_/dt = [ƛ * P_C_ * q] *(development of symptoms)* **(S1.5)**

- [ɣ_Y_ * Y_C_] *(recovery from symptomatic)*

d**R_C_**/dt = [ɣ_A_ * A_C_] *(recovery from symptomatic)* **(S1.6)**

- [ɣ_Y_ * Y_C_] *(recovery from asymptomatic)*
- [⍺ * R_C_] *(loss of immunity)*

### **Employee SEPAYDR:**

d**S_W_**/dt = - [p * β_W_ * S_W_ * (P_W_ + A_W_ + Y_W_) / N_W_] *(exposure from employees in the workplace)* **(S1.7)**

- [(1 - p) * β_C_ * S_W_ * (P_C_ + A_C_ + Y_C_) / (N_C_ + N_W_)] *(exposure from non-employees after work)*
- [(1 - p) * β_C_ * S_W_ * (P_W_ + A_W_ + Y_W_) / (N_C_ + N_W_)] *(exposure from employees in the community, after work)*
- [⍺ * R_W_] *(loss of immunity)*

d**E_W_**/dt = [p * β_W_ * S_W_ * (P_W_ + A_W_ + Y_W_) / N_W_] *(exposure from employees in the workplace)* **(S1.8)**

- [(1 - p) * β_C_ * S_W_ * (P_C_ + A_C_ + Y_C_) / (N_C_ + N_W_)] *(exposure from non-employees after work)*
- [(1 - p) * β_C_ * S_W_ * (P_W_ + A_W_ + Y_W_) / (N_C_ + N_W_)] *(exposure from employees in the community, after work)*
- [θ * E_W_] *(development of infectiousness)*

d**P_W_**/dt = [θ * E_W_] *(development of infectiousness)* **(S1.9)**

- [ƛ * P_W_ * (1 - q)] *(classification as persistently asymptomatic)*
- [ƛ * P_W_ * q] *(development of symptoms)*
- [**𝜏**_A_ * sens * P_W_] *(detection through asymptomatic testing)*

d**A_W_**/dt = [ƛ * P_W_* (1 - q)] *(classification as persistently asymptomatic)* **(S1.10)**

- [**𝛕**_A_ * sens * A_W_] *(detection through asymptomatic testing)*
- [ɣ_A_ * A_W_] *(recovery from asymptomatic, without detection)*

d**Y_W_**/dt = [ƛ * P_W_* q] *(development of symptoms)* **(S1.11)**

- [**𝜏**_Y_ * sens * Y_W_] *(detection through symptomatic testing)*
- [ɣ_Y_ * Y_W_] *(recovery from symptomatic, without detection)*

d**D_W_**/dt = [**𝜏**_A_ * sens * P_W_] *(detection from presymptomatic)* **(S1.12)**

- [**𝜏**_A_ * sens * A_W_] *(detection from asymptomatic)*
- [**𝜏**_Y_ * sens * Y_W_] *(detection from symptomatic)*
- [ɣ_D_ * D_W_] *(recovery, after detection)*

d**R_W_**/dt = [ɣ_A_ * A_W_] *(recovery from asymptomatic, without detection)* **(S1.13)**

- [ɣ_Y_ * Y_W_] *(recovery from symptomatic, without detection)*
- [ɣ_D_ * D_W_] *(recovery from detected)*
- [⍺ * R_W_] *(loss of immunity)*

Of note, the efficacy of any real-world testing program in finding those who are infected is attenuated by the sensitivity of the test being used. Thus, all testing rates (𝜏) in Equations S1.9 - S1.12 are multiplied by the sensitivity of the test (“sens”), which is typically below 1.0 [5].

In parallel to the differential equations for compartmental transitions, our model also estimates the total number of tests that are performed over the time period of interest. The test count includes tests performed due to reported symptoms, random monitoring testing of the population, and recovery testing that allows isolated individuals to return to the workplace. Estimating the number of tests of symptomatic individuals requires making an assumption about the proportion of non-cases that report symptoms each day (g parameter in S1.1 Table). Thus, this additional variable counting tests being performed is tracked as follows:

d**Tests**/dt = [**𝜏**_A_ * (S_W_ + E_W_ + P_W_ + A_W_ + R_W_)] *(asymptomatic testing)* **(S1.14)**

- [**𝜏**_Y_ * (Y_W_ + (S_W_ + E_W_ + P_W_ + A_W_ + R_W_) * g)] *(symptomatic testing)*
- ɣ_D_ * D_W_ * h *(tests required to return to work after isolation)*

Note that S1.14 Equation includes individuals in the recovered compartment (R_W_) in both symptomatic and asymptomatic testing. The recovered compartment includes both individuals who moved from detected to recovered (“known recovereds”), and those who moved from symptomatic or asymptomatic to recovered (“unknown recovereds”). Because the unknown recovereds cannot be distinguished in practice from the susceptible population, an employer cannot actually ever choose to exclude all recovered individuals from testing. While we could add here an additional compartment to separate known and unknown recovereds (thus allowing known recovereds to be excluded from testing), this adds complexity to the model that is only necessary for the secondary analysis of counting tests. Instead, we have chosen for simplicity of exposition here to include all recovered individuals in ongoing testing. Moreover, employers in practice may in fact choose such a conservative approach due to the unknowns regarding the degree of immunity conferred to individuals who have recovered from the virus, and also because there may be individuals who received a false positive result and so are incorrectly believed to be recovered from the virus.

The main analyses presented in this paper focus on the prevalence of cases of active infection among employees (that is, those that have the potential to be infectious if they interact with other individuals). This quantity is calculated as:

Prevalence of infection among employees = (P_W_ + A_W_ + Y_W_ + D_W_) / N_W_ **(S1.15)**

Prevalence among non-employees is calculated analogously to S1.15 Equation, though without the “Detected” compartment (since we are not modeling testing in the community).

For a given simulation run of the model, the initial state of the system is defined to match the current prevalence in the population of interest. To start, the size of the “Infected” compartment in the community sub-model is determined by the reported prevalence in the community. Next, recall that the model includes the option for the entire workforce to undergo initial testing prior to returning to the workplace. With a perfectly sensitive test, this approach would result in an initial employee prevalence of 0%. However, to account for imperfect test sensitivity,^19^ the expected initial employee prevalence can be calculated by multiplying the false negative rate of the test by the initial community prevalence:

Expected initial workplace prevalence = E[prv_Wi_] = prv_Ci_ * (1 - sens)  **(S1.16)**

S1.16 Equation is used to initialize the workplace prevalence value when initial testing is selected as part of the overall testing strategy. On the other hand, if the option for initial testing prior to returning to work is not selected, the workplace prevalence is simply initialized to be equal to the starting community prevalence (prv_Ci_). In either case, the initial infected (but not detected) employee population is distributed between the “Symptomatic” and “Asymptomatic” compartments, according to the parameter q. Any infected individuals who were identified during an initial testing process begin the simulation in the “Detected” compartment. For simplicity, all other compartments are initialized to occupancies of 0.

In the Results section, we present 3 case studies that demonstrate the flexibility of the “Community-Workplace” modeling framework. These case studies involve configuring the model to emulate an office workplace, a factory floor, and a university. S3 Table gives the values of the model parameters that are held constant for all case studies, and in all follow-up simulations except where otherwise noted. In the main text, we described the choices of the R0_W_ and p parameters that are varied to capture an idealized portrayal of each case study. Thus, the parameters for each of the case studies are fully described by these case-specific values alongside the values in S3 Table.

**S3 Table: Model parameters held constant for all case studies. All model parameters not listed here or in the main text can be calculated from these values using the formulae in S2 Table. See S2 for a discussion of how these values were selected.**

| **Model Concept** | **Notation** | **Value** |
| --- | --- | --- |
| Length of simulation (days) | L | 100 |
| “Workplace” population size (i.e., number of employees) | N_W_ | 1,000 |
| “Community” population size (i.e., number of non-employees) | N_C_ | 500,000 |
| Prevalence in the community at the beginning of the simulation | prv_Ci_ | 1% |
| Proportion of cases that develop symptoms | q | 60% |
| Proportion of non-cases that report symptoms each work-day | g | 0.1% |
| Average days taken to develop infectiousness after first exposure | Δ_infectious_ | 4 |
| Average days taken to recover from onset of infectiousness | Δ_recover_ | 10 |
| Average days taken to develop symptoms after becoming infectious | Δ_symptoms_ | 3 |
| Days required in isolation if tested positive | Δ_isolation_ | 7 |
| Average days of immunity after recovering | Δ_immunity_ | Infinity |
| Basic virus reproduction number in the community | R0_C_ | 1.3 |
| Test sensitivity | sens | 0.8 |
| Average number of tests required to return to work after infection | h | 1 |
