# Supplementary figures and images for: A holistic approach for suppression of COVID-19 spread in workplaces and universities
Source: PLoS One. 2021 Aug 12;16(8):e0254798. doi: 10.1371/journal.pone.0254798 (PMC8360595; doi:10.1371/journal.pone.0254798)

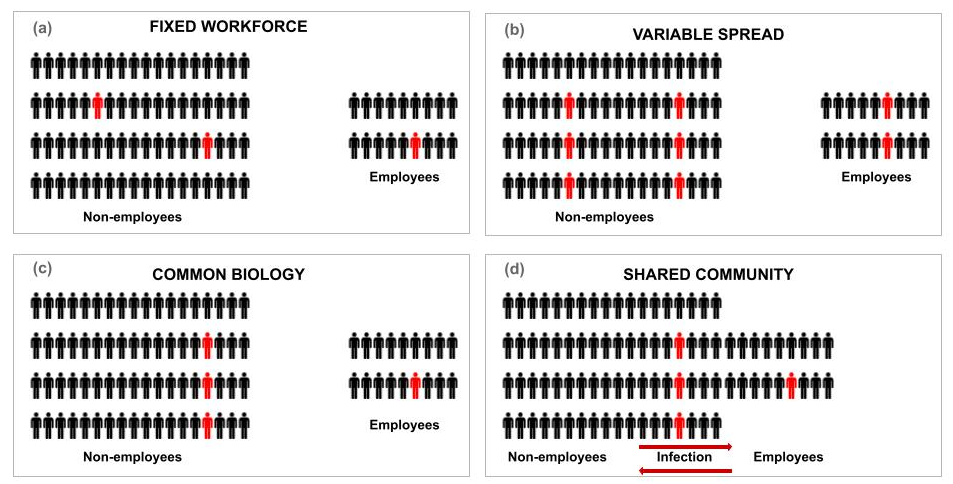

Supplement: S1 File — (ZIP) [file pone.0254798.s001.zip › S1_Fig.tiff]

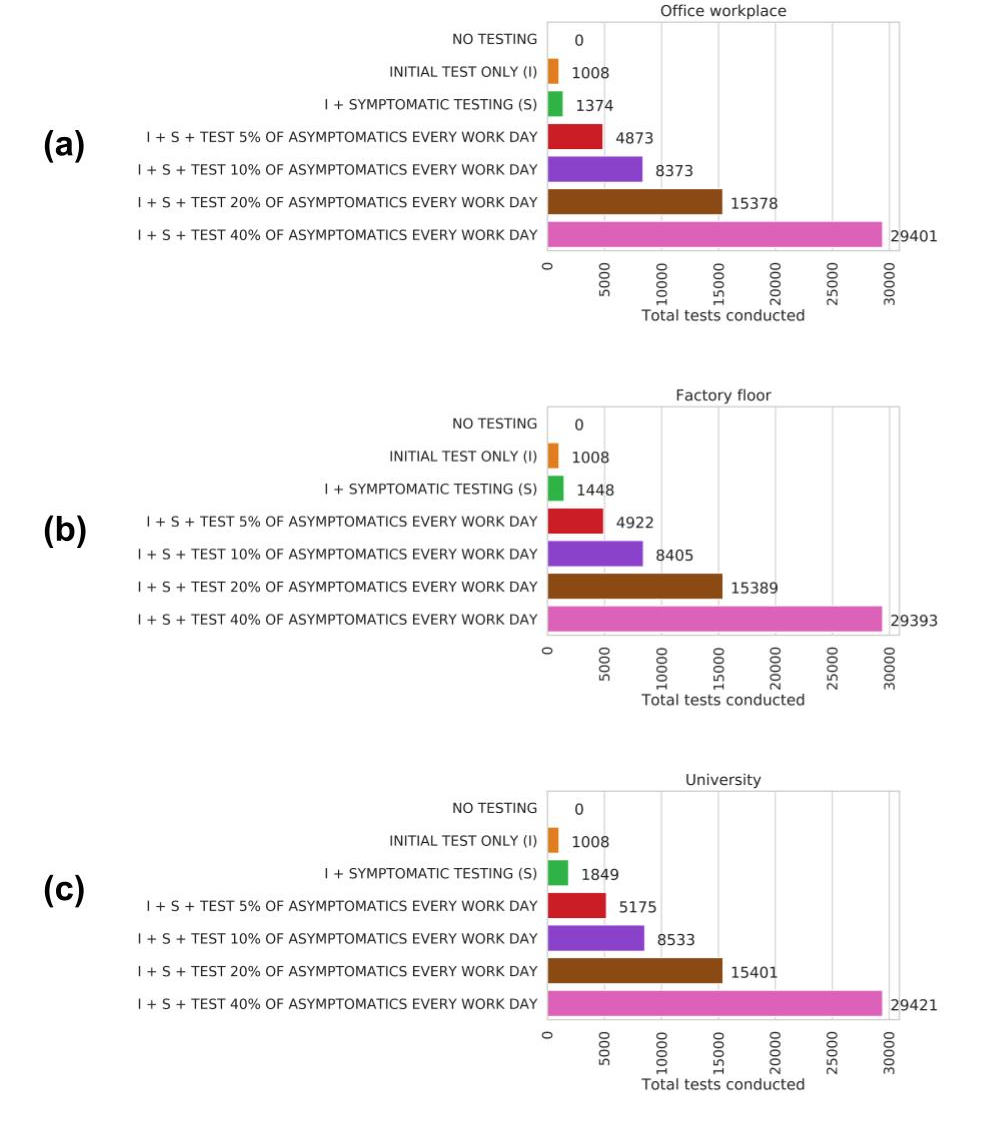

Supplement: S3 File — (ZIP) [file pone.0254798.s003.zip › S7_Fig.tiff]

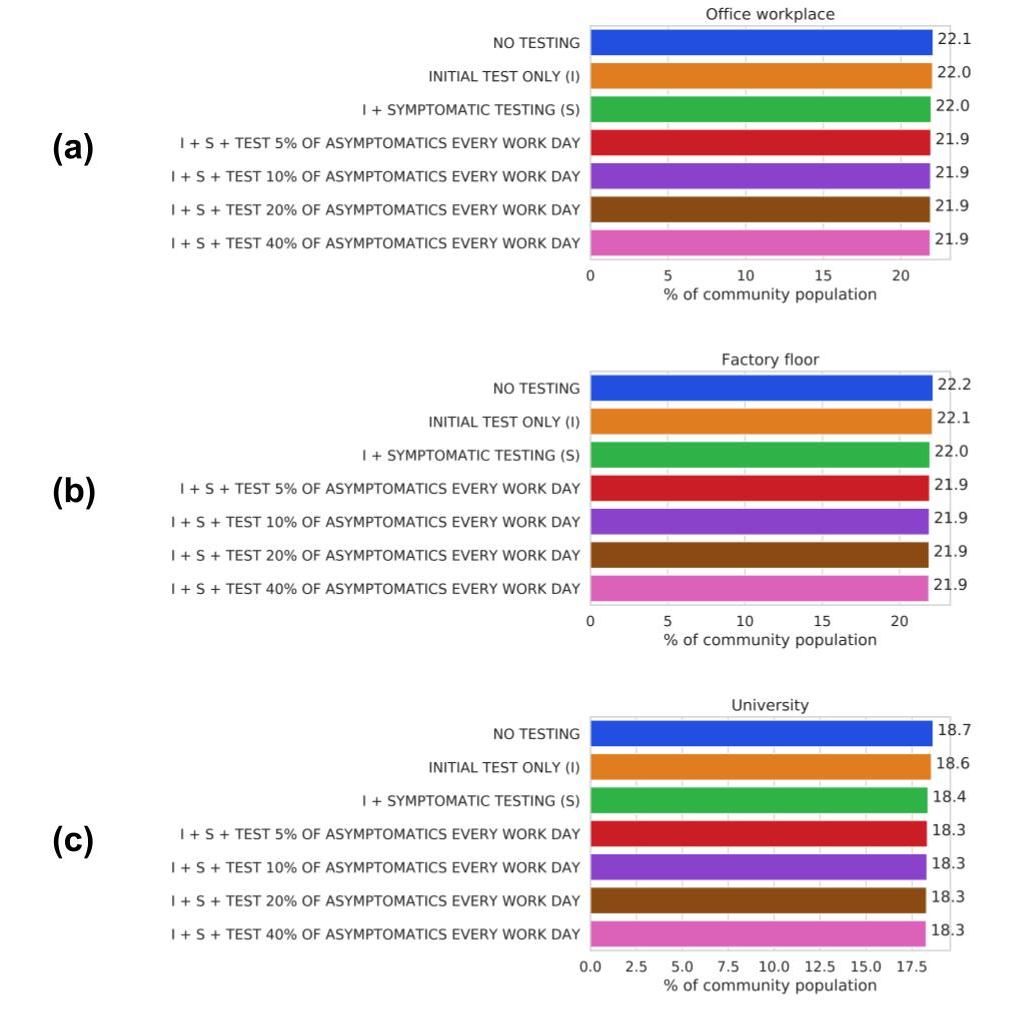

Supplement: S3 File — (ZIP) [file pone.0254798.s003.zip › S6_Fig.tiff]

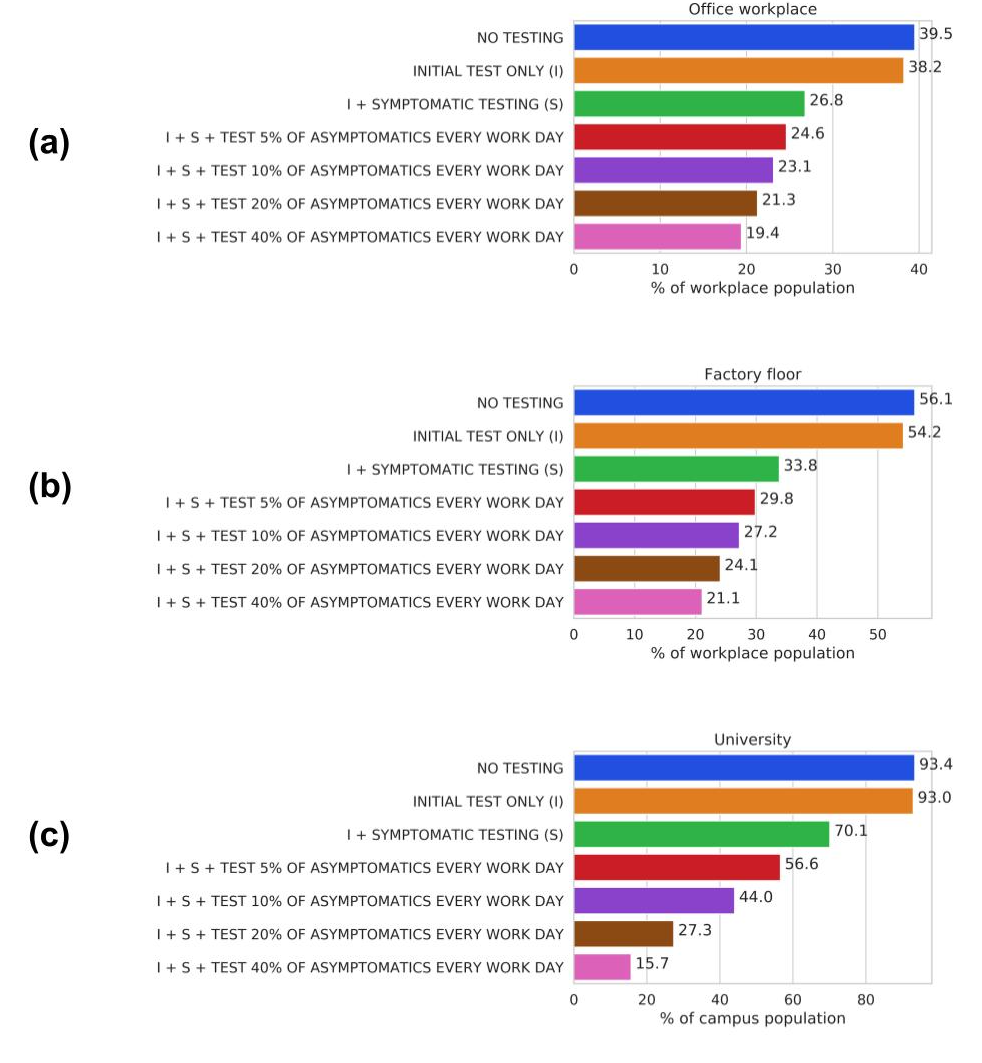

Supplement: S3 File — (ZIP) [file pone.0254798.s003.zip › S5_Fig.tiff]

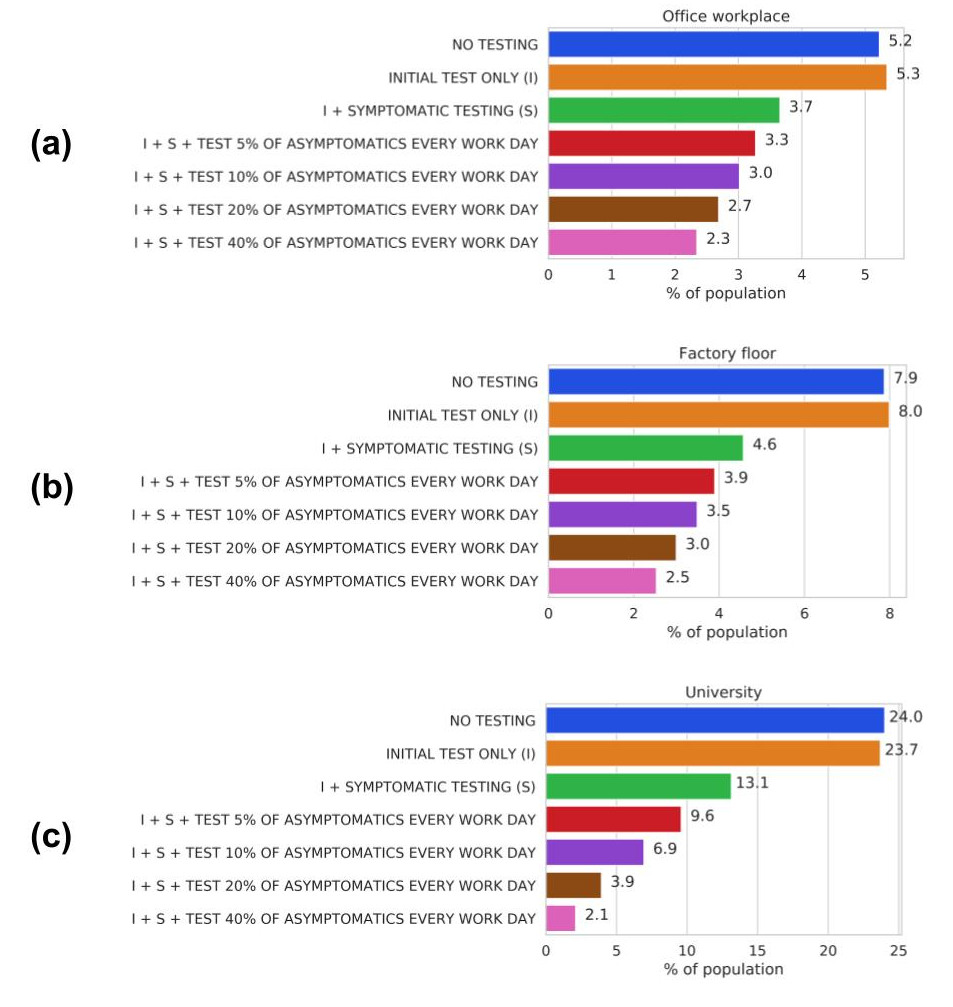

Supplement: S3 File — (ZIP) [file pone.0254798.s003.zip › S4_Fig.tiff]
