# Supplementary material for: A holistic approach for suppression of COVID-19 spread in workplaces and universities
Source: PLoS One. 2021 Aug 12;16(8):e0254798. doi: 10.1371/journal.pone.0254798 (PMC8360595; doi:10.1371/journal.pone.0254798)
Supplement: S2 File — (DOCX) [file pone.0254798.s002.docx]

# **S2 File: Estimation of community and workplace model parameters**

The compartmental model presented has a large number of parameters. Some of these parameters are related to epidemiological characteristics of the SARS-CoV-2 virus and the associated COVID-19 disease. Because this virus and the disease that it can cause are not yet fully understood, there is uncertainty in the values of these parameters. Other parameters are related to the initial prevalence and transmission rates in the populations of interest. Translating a realistic scenario into the appropriate values of these parameters is challenging, as there does not yet exist enough data for us to accurately estimate how different workplace characteristics and policies (e.g., employee density, mask-wearing, and hand-washing) translate into epidemiological concepts such as R0. To ensure that the uncertainty in the value of the model parameters is reflected in the model output, we suggest performing a probabilistic sensitivity analysis, varying parameters over their expected range [6].

The following sections specify how the default values of each model parameter are currently being estimated.

## **Initial community prevalence**

This parameter should reflect the total number of active cases in the community. Most US states and counties report the number of confirmed cases that are logged each day through publicly available channels. However, due to variation in testing availability and criteria in different geographic areas, it is not straightforward to translate from reported cases to active cases of infection [7-9]. Therefore, this is still an area of active exploration in the research community. The value used for initial community prevalence in our case studies is based on an average value for the estimated prevalence in US states. These estimates were initially obtained from a publicly available model ([covid19-projections.com](http://covid19-projections.com)) that fits an SIR-style model to reported case numbers and death rates to obtain estimated current prevalence for a geographic area of interest [10]; note that this web server has stopped driving prevalence values and thus we used a conservative estimate of 1% for the generic examples considered here. We found that lowering this parameter in the community from 1% to, e.g., 0.5%, simply somewhat delayed the timing of the prevalence trajectories rather than qualitatively changing their properties, in particular since we use R0_C_ > 1.

## **Community R0 (R0_C_)**

Similarly, the estimate of the current R0 in specific geographic areas is best obtained using a model that incorporates multiple sources of data, including reported case numbers and death rates. The value of this parameter used in our case studies is obtained from the same model we used for community prevalence, and also from the Rt.live model [10, 11].

## **Workplace R0 (R0_W_)**

Estimating an appropriate value of R0 for a specific type of workplace is another area of active research. Currently, values of this parameter are informed by looking at existing studies of COVID-19 [7, 8] and tracking of outbreaks within populations of interest [13].

## **Proportion of time spent at work**

The proportion of time that employees spend at work determines the amount of spread between the community and the workplace. In workplaces where employees typically work a 40 hour week, we set this parameter to 33%. This assumes that the employee is spending two-thirds of their time as a member of the wider community. This includes time outside of work during the week, as well as time over the weekend. For the “University” case study, we consider that many students live in dorms on campus, attend classes together, and eat most of their meals in on-campus dining halls. As such, the default value for “time spent at work” in the “University” case study is set to 70%.

## **The time course of disease**

Estimates of the time between exposure to the virus, development of infectiousness, and development of symptoms are based on CDC guidance, as well as values used in similar studies [3, 12, 14]. We use slightly more conservative values, in that we assume in an average of 4 days between exposure and the virus becoming detectable (and infectiousness). Even if the full population is tested each day, infected individuals who are in this latent period will not be detected. Similarly, we assume an average of 3 days between the development of infectiousness and the development of symptoms. This is an increase on the estimate of 2 days seen in other studies [3, 14], meaning that infected individuals will remain in the workplace for a longer period of time. The choice of these parameters ensures that we err of the side of being slightly pessimistic about outcomes within a population.

## **Proportion of cases with symptoms**

The model parameter q indicates the proportion of cases that are expected to self-report symptoms. The current best estimate from the CDC [15] is that 60% of COVID-19 cases will develop symptoms; we thus use this value.

## **Lapsing of immunity (⍺)**

The question of immunity after infection is still an area of research [16]. Because the simulations conducted by our model are on a relatively short time scale (100 days), we currently assume that all recovered individuals maintain immunity to further infection for the duration of the simulation, so the parameter ⍺ is set to 0. Understanding the appropriate value for ⍺ will be critical to ensure that populations who have been infected and have recovered, as well as populations who have been vaccinated for COVID-19, can be effectively incorporated into the model.

## **Infectiousness of asymptomatic individuals relative to symptomatic individuals**

The model presented assumes that asymptomatic individuals are equally infectious as symptomatic individuals. However, the CDC believes there is some evidence that asymptomatic individuals are somewhat less infectious than symptomatics [15], and a difference in relative infectiousness is actually built into some published models [17]. While adding this extension to our model would not be technically difficult, we feel that assuming equal infectiousness is the correct choice for our use case. In particular, assuming equal infectiousness gives a conservative (pessimistic) view of the potential spread within a workplace if asymptomatic individuals are undetected, ensuring that the model does not give employers an overly optimistic picture of the likely trajectory of an epidemic within their workplace.
