# Supplementary material for: A holistic approach for suppression of COVID-19 spread in workplaces and universities
Source: PLoS One. 2021 Aug 12;16(8):e0254798. doi: 10.1371/journal.pone.0254798 (PMC8360595; doi:10.1371/journal.pone.0254798)
Supplement: S3 File — (ZIP) [file pone.0254798.s003.zip › S3_File.docx]

# **S3 File: Case study results**

See S4 – S7 Figs for results.

Figure S4: Peak workplace prevalence (as percentage) under a range of testing strategies, for (a) Office workplace, (b) Factory floor, (c) University

Figure S5: Percentage of workforce population infected over the entire simulation (cumulative prevalence) under a range of testing strategies, for (a) Office workplace, (b) Factory floor, (c) University.

Figure S6: Percentage of community population infected over the entire simulation (cumulative prevalence) under a range of testing strategies, for (a) Office workplace, (b) Factory floor, (c) University.

Figure S7: Estimated total workplace tests conducted over the 100 day simulation under a range of testing strategies, for (a) Office workplace, (b) Factory floor, (c) University.
