# Supplementary material for: A holistic approach for suppression of COVID-19 spread in workplaces and universities
Source: PLoS One. 2021 Aug 12;16(8):e0254798. doi: 10.1371/journal.pone.0254798 (PMC8360595; doi:10.1371/journal.pone.0254798)
Supplement: S4 File — (DOCX) [file pone.0254798.s004.docx]

# **S4 File: References**

1. Guo H, Li MY, Shuai Z. Global stability of the endemic equilibrium of multigroup SIR epidemic models. Canadian applied mathematics quarterly. 2006;14(3):259-84.
2. Magal P, Seydi O, Webb G. Final size of a multi-group SIR epidemic model: Irreducible and non-irreducible modes of transmission. Mathematical biosciences. 2018 Jul 1;301:59-67.
3. Martin N, Schooley RT, De Gruttola V. Modelling testing frequencies required for early detection of a SARS-CoV-2 outbreak on a university campus. medRxiv. 2020 Jan 1.
4. Sethuraman N, Jeremiah SS, Ryo A. Interpreting diagnostic tests for SARS-CoV-2. Jama. 2020 May 6.
5. Zhen W, Smith E, Manji R, Schron D, Berry GJ. Clinical evaluation of three sample-to-answer platforms for detection of SARS-CoV-2. Journal of clinical microbiology. 2020 Jul 23;58(8).
6. Lopman B, Liu C, Le Guillou A, Handel A, Lash TL, Isakov A, Jenness S. A model of COVID-19 transmission and control on university campuses. medRxiv. 2020 Jan 1.
7. Benatia D, Godefroy R, Lewis J. Estimating COVID-19 prevalence in the United States: A sample selection model approach. medRxiv. 2020 May 18.
8. Lu FS, Nguyen A, Link N, Santillana M. Estimating the prevalence of COVID-19 in the United States: three complementary approaches. medRxiv. 2020 Jun 18.
9. Griffith G, Morris TT, Tudball M, Herbert A, Mancano G, Pike L, Sharp GC, Palmer TM, Smith GD, Tilling K, Zuccolo L. Collider bias undermines our understanding of COVID-19 disease risk and severity. medRxiv. 2020 Jan 1.
10. Gu Y. COVID-19 Projections Using Machine Learning [Internet]. Cited November 16, 2020. Available at: https://covid19-projections.com/
11. Systrom K, Vladeck T, Krieger M. R_t_ COVID-19 [Internet]. Cited November 16, 2020. Available at: https://rt.live/
12. Paltiel AD, Zheng A, Walensky RP. Assessment of SARS-CoV-2 screening strategies to permit the safe reopening of college campuses in the United States. JAMA network open. 2020 Jul 1;3(7):e2016818-.
13. The New York Times. Tracking the Coronavirus at U.S. Colleges and Universities [Internet]. The New York Times. Cited November 16, 2020. Available at: https://www.nytimes.com/interactive/2020/us/covid-college-cases-tracker.html
14. Chin ET, Huynh BQ, Murrill M, Basu S, Lo NC. Frequency of routine testing for COVID-19 in high-risk environments to reduce workplace outbreaks. medRxiv. 2020 Jan 1.
15. CDC. Pandemic Planning Scenarios [Internet]. Centers for Disease Control and Prevention. Updated September 10, 2020. Cited November 16, 2020. Available at: https://www.cdc.gov/coronavirus/2019-ncov/hcp/planning-scenarios.html
16. Kirkcaldy RD, King BA, Brooks JT. COVID-19 and Postinfection Immunity: Limited Evidence, Many Remaining Questions. Jama. 2020 Jun 9;323(22):2245-6.
17. Hao X, Cheng S, Wu D, Wu T, Lin X, Wang C. Reconstruction of the full transmission dynamics of COVID-19 in Wuhan. Nature. 2020 Aug;584(7821):420-4.
