# Supplementary material for: A holistic approach for suppression of COVID-19 spread in workplaces and universities
Source: PLoS One. 2021 Aug 12;16(8):e0254798. doi: 10.1371/journal.pone.0254798 (PMC8360595; doi:10.1371/journal.pone.0254798)
Supplement: S1 Table — (PDF) [file pone.0254798.s005.pdf]

S1 Table. Notation and formulae for parameters used in the "Community-Workplace" model.

Model Concept	Notation
Length of modeling simulation (days)	L
Time (timepoint of the simulation)	t
"Workplace" population size (i.e., number of employees)	N_W
"Community" population size (i.e., number of non-employees)	N_C
Initial disease prevalence in the community (at the beginning of the simulation)	prv_{Ci}
Initial disease prevalence in the workplace (at the beginning of the simulation)	prv_{Wi}
Proportion of time employees spend at work (interacting only among themselves)	p
Proportion of cases that develop symptoms (vs. being "asymptomatic")	q
Proportion of non-cases that report symptoms each work-day	g
Average days it takes to develop infectiousness after infection-causing exposure to virus	$\Delta_{infectious}$
Rate of development of infectiousness (= 1 / $\Delta_{infectious}$)	θ
Average days of being infectious. Equivalent to the average days taken to recover from onset of infectiousness.	$\Delta_{recover}$
Rate of recovery from onset of infectiousness (= 1 / $\Delta_{recover}$)	γ_I
Average days taken to develop symptoms after becoming infectious (for non-"asymptomatic"s)	$\Delta_{symptoms}$
Rate of symptom development for infected individuals who become symptomatic (= 1 / $\Delta_{symptoms}$)	λ
Rate of recovery from onset of symptoms (= 1 / ($\Delta_{recover}$ - $\Delta_{symptoms}$))	γ_Y
Rate of recovery from moving into asymptomatic compartment (= 1 / ($\Delta_{recover}$ - $\Delta_{symptoms}$))	γ_A
Days required in isolation if tested positive	$\Delta_{isolation}$
Rate of movement back to work after being detected (= 1 / $\Delta_{isolation}$)	γ_D
Average days of immunity after recovering	$\Delta_{immunity}$
Rate of loss of immunity (= 1 / $\Delta_{immunity}$)	α
Basic virus reproduction number (i.e., the mean number of people in a fully susceptible population that are infected with SARS-CoV-2 by a single infected person) in the workplace	$R0_W$
Basic virus reproduction number (i.e., the mean number of people in a fully susceptible population that are infected with SARS-CoV-2 by a single infected person) in the community	$R0_C$
Transmission rate in the workplace (= $R0_W$ * γ_I)	β_W
Transmission rate in the community (= $R0_C$ * γ_I)	β_C
Proportion of the asymptomatic workforce population tested each day	τ_A

Proportion of the symptomatic workforce population tested each day	τ_Y
Test sensitivity (probability of an infected individual's test being positive)	sens
Average number of tests required to return to work after infection (i.e., "testing out of isolation")	h
