# Supplementary material for: A holistic approach for suppression of COVID-19 spread in workplaces and universities
Source: PLoS One. 2021 Aug 12;16(8):e0254798. doi: 10.1371/journal.pone.0254798 (PMC8360595; doi:10.1371/journal.pone.0254798)
Supplement: S2 Table — All model parameters not listed here or in the main text can be calculated from these values using the formulae in S1 Table. See S2 File for a discussion of how these values were selected. (PDF) [file pone.0254798.s006.pdf]

S2 Table. Model parameters held constant for all case studies. All model parameters not listed here or in the main text can be calculated from these values using the formulae in Table S1.1. See Supplement S2 for a discussion of how these values were selected.

**Model Concept**	**Notation**	**Value**
Length of simulation (days)	L	100
"Workplace" population size (i.e., number of employees)	N_W	1,000
"Community" population size (i.e., number of non-employees)	N_C	500,000
Prevalence in the community at the beginning of the simulation	prv_{Ci}	1%
Proportion of cases that develop symptoms	q	60%
Proportion of non-cases that report symptoms each work-day	g	0.1%
Average days taken to develop infectiousness after first exposure	$\Delta_{infectious}$	4
Average days taken to recover from onset of infectiousness	$\Delta_{recover}$	10
Average days taken to develop symptoms after becoming infectious	$\Delta_{symptoms}$	3
Days required in isolation if tested positive	$\Delta_{isolation}$	7
Average days of immunity after recovering	$\Delta_{immunity}$	Infinity
Basic virus reproduction number in the community	$R0_C$	1.3
Test sensitivity	sens	0.8
Average number of tests required to return to work after infection	h	1
