# Supplementary material for: A holistic approach for suppression of COVID-19 spread in workplaces and universities
Source: PLoS One. 2021 Aug 12;16(8):e0254798. doi: 10.1371/journal.pone.0254798 (PMC8360595; doi:10.1371/journal.pone.0254798)
Supplement: Attachment — Submitted filename: Response to Reviewers.pdf [file pone.0254798.s007.pdf]

Dear Dr. Heber, the PLOS ONE Editorial team, and Reviewer #1,

Thank-you for the review of our manuscript entitled 'A holistic approach for suppression of COVID-19 spread in workplaces and universities', and the opportunity to resubmit our revised manuscript.

We appreciate the careful review of our manuscript, and the thoughtful comments around implications of the model results. We have added to the manuscript to acknowledge these implications.

The reviewer mentioned the many policy implications for our model's test results, including on increasing testing capabilities. The goal of our manuscript is not to prescribe an optimal testing strategy, as through our work with various employers we have found that the cost-benefit trade off being performed varies significantly across different industries and, more generally, across different employers. We have added commentary in the Discussion section to note that our focus is on presenting a model for others to use and interpret according to their specific use case, rather than on presenting specific model results that prescribe the best decisions to make moving forward. We hope that this addresses the reviewer's point satisfactorily.

The reviewer also mentioned that the model results may have implications for diversification of testing tools, such as the use of rapid diagnostic testing. The model that we present has a large number of parameters to explore, and to keep the scope of the manuscript manageable we have chosen to focus on specifically varying the parameters that define the characteristics of the workplace environment, and on the frequency of testing. We agree with the reviewer's insight that this model could be used to explore the impact of different testing types, and have added this as a note in the Discussion section to highlight this use of the model.

The reviewer notes that the model results highlight social inequalities in health, in that it shows a higher peak prevalence is likely in an employee population that works in a factory setting compared to an office workplace. We agree that the disparate impact of the virus across social groups is of key importance and concern. We have added language in the Discussion section to acknowledge this result, and highlight that this should be carefully considered by groups determining the appropriate testing strategy to employ. We have also noted the importance of testing availability outside a workplace setting (i.e., testing provided by the state or county) in ensuring that all members of the community are able to monitor their health. We thank the reviewer for highlighting this important point, and hope that the insights provided in our manuscript can help both workplaces and policy makers to make informed decisions about how their testing programs will impact the population that they are serving

Another point that was raised by the reviewer is that the emergence of new viral variants, with different transmission characteristics, will impact the model results. We agree that results will

certainly be impacted by changes in transmissibility of the virus, and also note that the vaccination rollout will impact the spread of the virus. The main parameters in the model that captures the virus transmissibility are $R0_W$ and $R0_C$. As we discuss in Supplement S2, the value used for $Ro_C$ is obtained from publicly available modeling that identifies the value that best fits the trend in the number of reported cases. Both new variants and vaccination amount will impact the number of cases being reported, so we believe that the model is able to capture the impact of these key changes without them being explicitly included in the transmission model.

We also thank the reviewer for identifying the missing legend label for Figure 2. This has been updated. Additionally, we have updated our language to more accurately refer to ‘physical distancing’ rather than ‘social distancing’.

Finally, we have updated our manuscript formatting and file naming to better comply with requirements. We have also amended our Funding Statement and Competing Interests Statement, and these are included in the Cover Letter, as requested.

We look forward to your response.

Regards,
Sarah F. Poole
Corresponding Author
sarahfpoole@verily.com
